# Cell Therapy in Veterinary Medicine as a Proof-of-Concept for Human Therapies: Perspectives From the North American Veterinary Regenerative Medicine Association

**DOI:** 10.3389/fvets.2021.779109

**Published:** 2021-11-30

**Authors:** Boaz Arzi, Tracy L. Webb, Thomas G. Koch, Susan W. Volk, Dean H. Betts, Ashlee Watts, Laurie Goodrich, Michael S. Kallos, Amir Kol

**Affiliations:** ^1^Department of Surgical and Radiological Sciences, School of Veterinary Medicine, University of California, Davis, Davis, CA, United States; ^2^Veterinary Institute for Regenerative Cures, School of Veterinary Medicine, University of California, Davis, Davis, CA, United States; ^3^Department of Clinical Sciences, Colorado State University, Fort Collins, CO, United States; ^4^Department of Biomedical Sciences, Ontario Veterinary College, University of Guelph, Guelph, ON, Canada; ^5^Department of Clinical Sciences and Advanced Medicine, University of Pennsylvania School of Veterinary Medicine, Philadelphia, PA, United States; ^6^Department of Physiology and Pharmacology, Schulich School of Medicine and Dentistry, The University of Western Ontario, London, ON, Canada; ^7^Department of Large Animal Clinical Sciences, Veterinary Medicine and Biological Sciences, Texas A&M University, Killeen, TX, United States; ^8^Department of Chemical and Petroleum Engineering, Schulich School of Engineering, and Biomedical Engineering Graduate Program, University of Calgary, Calgary, AB, Canada; ^9^Department of Pathology, Microbiology, and Immunology, School of Veterinary Medicine, University of California, Davis, Davis, CA, United States

**Keywords:** companion animals, stem cell, naturally occurring, One Health, clinical trial, therapy

## Abstract

In the past decade, the potential to translate scientific discoveries in the area of regenerative therapeutics in veterinary species to novel, effective human therapies has gained interest from the scientific and public domains. Translational research using a One Health approach provides a fundamental link between basic biomedical research and medical clinical practice, with the goal of developing strategies for curing or preventing disease and ameliorating pain and suffering in companion animals and humans alike. Veterinary clinical trials in client-owned companion animals affected with naturally occurring, spontaneous disease can inform human clinical trials and significantly improve their outcomes. Innovative cell therapies are an area of rapid development that can benefit from non-traditional and clinically relevant animal models of disease. This manuscript outlines cell types and therapeutic applications that are currently being investigated in companion animals that are affected by naturally occurring diseases. We further discuss how such investigations impact translational efforts into the human medical field, including a critical evaluation of their benefits and shortcomings. Here, leaders in the field of veterinary regenerative medicine argue that experience gained through the use of cell therapies in companion animals with naturally occurring diseases represent a unique and under-utilized resource that could serve as a critical bridge between laboratory/preclinical models and successful human clinical trials through a One-Health approach.

## Introduction

For centuries, starting at a time when physicians cared for both human patients and their animals, human and veterinary health have been intertwined. Veterinarians, physicians, and other scientific health and environmental professionals, in an initiative now referred to as “One Health,” have started capitalizing on this approach to improve the lives of all species (1–5). Due to shared commonalities such as pathophysiology of specific disease states, co-morbidities, and extrinsic factors, which may influence treatment outcomes in people and animals, a One Health approach has the potential to better predict therapeutic success and efficiently translate promising medical advances in human and veterinary patients (2). Notably, the field of regenerative medicine can benefit from the incorporation of companion animals in the assessment of novel therapies and thereby change the trajectory of care for human and veterinary patients (4, 6–8).

The significant number of failures of phase II and III human clinical trials in reproducing the success of preclinical trials has raised awareness of model fidelity (9–11). Rodent models play an invaluable role in biomedical research; however, awareness of the beneficial role of companion animals in translational research is increasing. Naturally occurring diseases in these species caused by complex interactions between multiple genes and environmental factors may provide several distinct advantages over induced models of disease for translational studies and for discovery science for which acceptable models are lacking (9). Currently, there are at least 462 canine, 223 feline, and 132 equine potential models of human diseases associated with Mendelian traits (www.omia.org) and many more that are not associated with specific genetic causes that provide critical model features of high fidelity.

**Figure 1:**
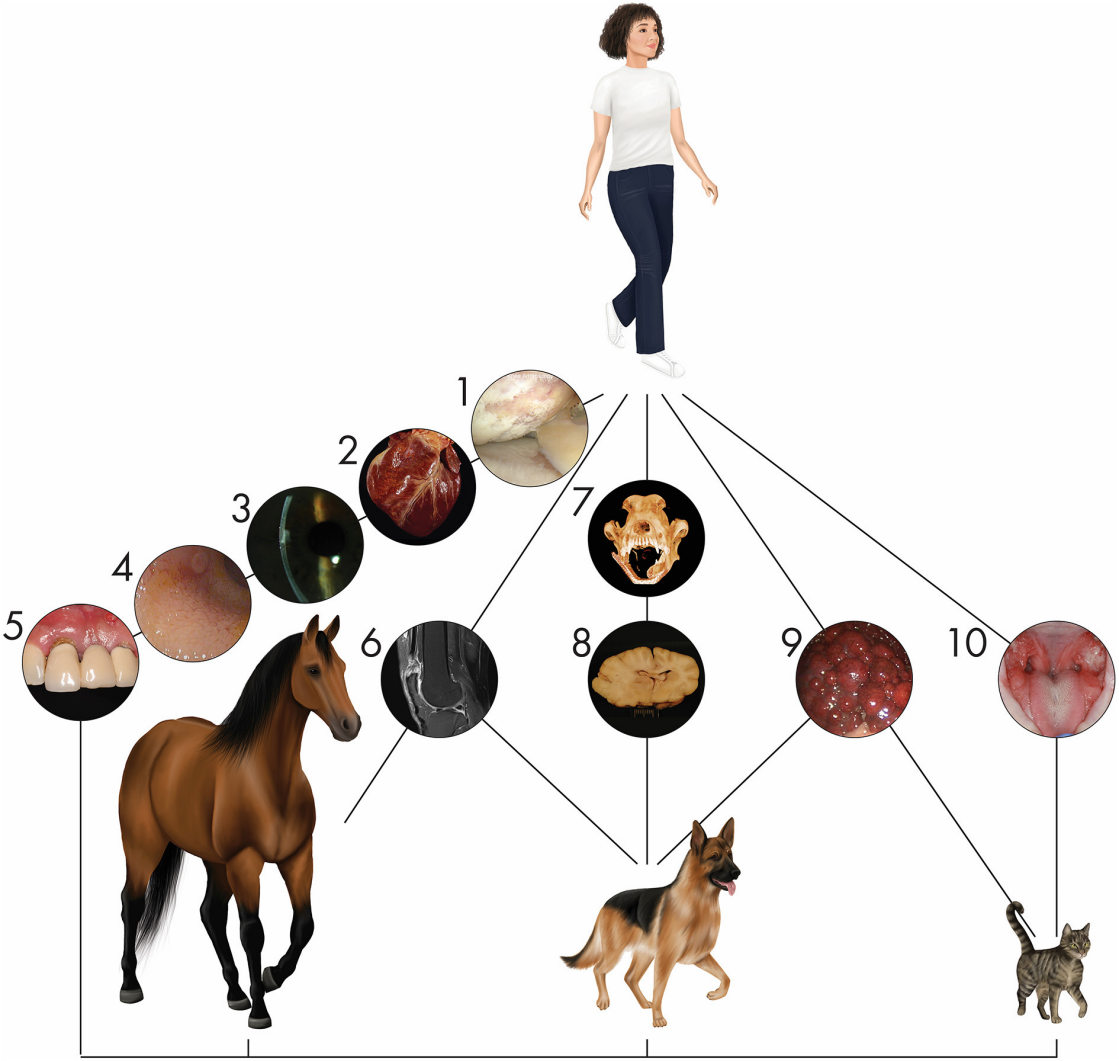
Naturally occurring diseases are shared between companion animal and human patients. Representative diseases in different organ systems affecting multiple species are depicted. (1) Arthroscopic image of a human knee joint affected by severe osteoarthritis. Osteoarthritis is one of the most common orthopedic conditions affecting humans and companion animals. (2) Heart of a dog affected by myocardial disease. Specific cardiac disorders, well known in humans, are also seen in veterinary patients. (3) Corneal endothelial dystrophy in the eye of a person is similar to that seen in dogs. (4) Gastroduodenal endoscopy in a cat reveals inflammatory bowel disease. Its appearance and pathogenesis have extensive similarities with the human condition. (5) Periodontal disease in a person is similar to the disease seen in dogs and cats with prevalence positively associated with increasing age. (6) Tendon injury is a common sport injury, whether the athlete/patient is human (seen here), horse, or dog. (7) Mandibular reconstruction to repair critical-size bone defects using regenerative approaches in dogs are informing similar approaches in humans (12–14). (8) Inflammatory brain disease in dogs serves as a model for this human condition in clinical trials using stem cells. (9) A cirrhotic liver arising from chronic hepatitis in a dog shares parallels with the human condition. (10) Stem cell therapy for the treatment of chronic gingivostomatitis in cats, a chronic oral mucosal inflammatory disorder, provides hope for successful treatment of similar human disorders such as lichen planus through immunomodulation (8, 15, 16).

Companion animals are relatively outbred with a longer life-span and larger size permitting diagnostic and treatment options that cannot be performed in rodent models and with basic biochemical and physiological processes which more closely resemble those in humans when compared to rodents (Figure 1) (17). Imaging and longitudinal biologic sampling may not be feasible yet in rodent models, which is particularly important in monitoring for clinical efficacy and side effects associated with novel therapies to minimize veterinary and human patient risk. Furthermore, companion animals are exposed to external and environmental factors, which influence disease development, progression, impact of therapeutics, and subject these patients to traumatic injury in a manner similar to human patients (2, 5). Critical to the ongoing success and protection of these valuable models is an increased demand for sophisticated, cutting-edge care for companion animals and the resulting surge in veterinary clinical trials. These veterinary clinical trials not only provide valuable insight into efficacy and safety of therapies for extrapolation in humans but improve the standard of care for veterinary patients. Given the value of a One Health approach, we argue that regenerative therapies in veterinary species in translational and clinical practice are beneficial for the efficient advancement of the field. The goal of this commentary is to review the use of various cell types and their derived products for regenerative medicine applications in companion animals including practices from cell processing and development to distribution and administration to assist in improved translational applications.

## Cell Types and Cell-Based Products

### Mesenchymal Stromal Cells

The most well-studied cell type in veterinary medicine, *ex-vivo* expanded mesenchymal stromal cells (MSCs) are plastic-adherent cells that have regenerative and immunomodulatory properties *via* paracrine activity (18). When maintained in serum-supplemented media, plastic-adherent MSCs replicate quickly and maintain anti-inflammatory, angiogenic, and regenerative properties (18). The source tissue should be identified when discussing MSCs because cells from varying sources have differing properties. Well-accepted acronyms for tissue of origin include bone marrow-derived (BM-MSC), adipose-derived (AD-MSC), and umbilical cord tissue-derived (UC-MSC) (19). MSCs derived from all of these tissue types have been investigated in companion animal models for the treatment of a variety of diseases, from osteoarthritis to inflammatory bowel disease (IBD). To best use data from these spontaneous animal models, both the disease and MSCs should be thoroughly characterized. MSCs from some animal species can have variable properties when compared to other species such as humans, and any differences should be evaluated for their potential impact on outcomes (20). When using MSCs, the International Society for Cell and Gene Therapy (ISCT) recommends that an array of functional assays be included that reflect the expected functional benefit when used therapeutically (19).

### Specialized Immune Cells

**Figure 2:**
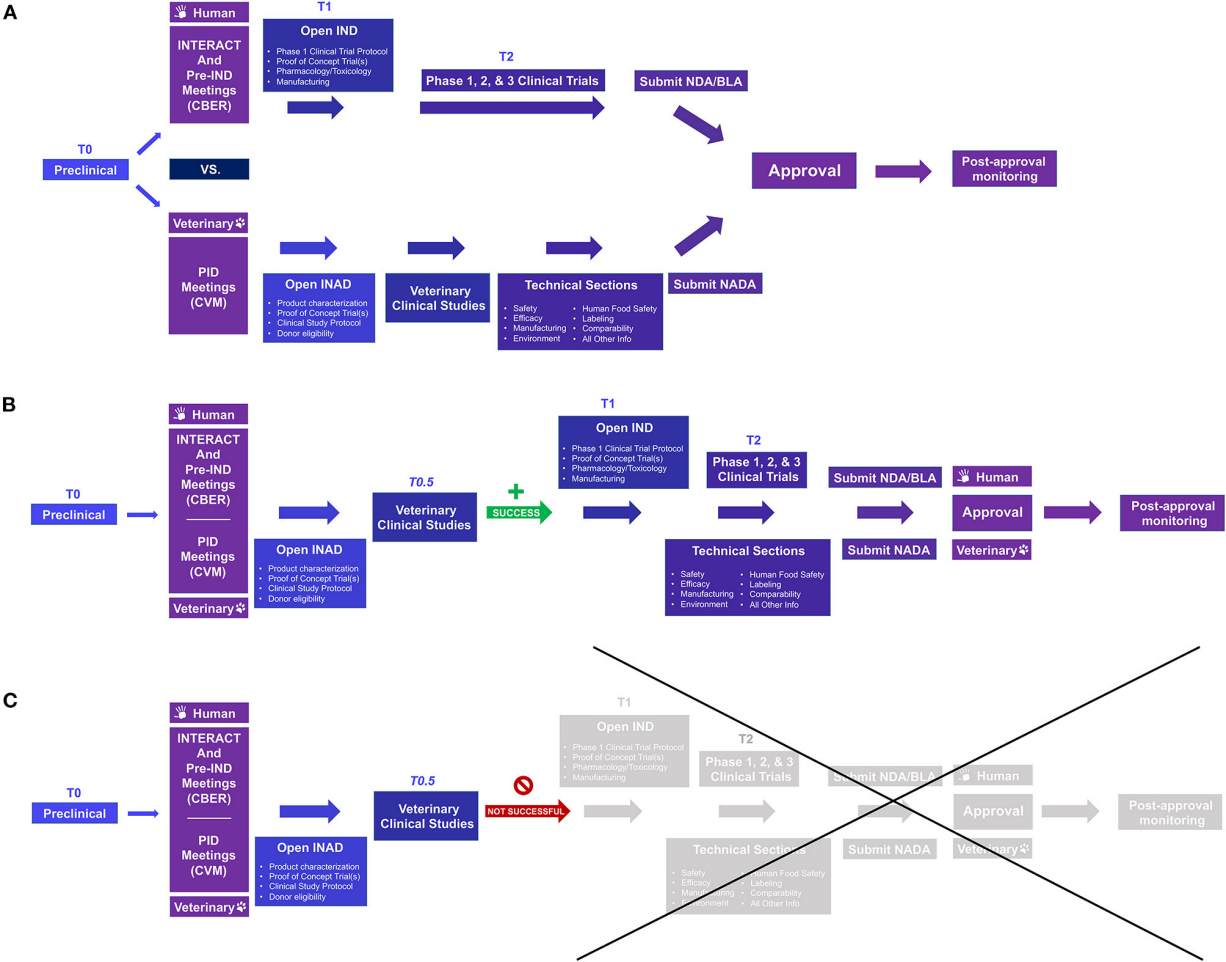
Comparison of the US FDA approval process for human and veterinary cell-based products and opportunities to use data from naturally occurring models for dual species product approval. **(A)** Prior to conducting clinical investigations, an Investigational New Drug (IND) or Investigational New Animal Drug (INAD) application is filed with FDA Center for Biologics Evaluation and Research (CBER) or Center for Veterinary Medicine (CVM), respectively. For efficiency, INitial Targeted Engagement for Regulatory Advice on CBER producTs (INTERACT) and pre-IND meetings on the human side and Pre-Investigation Development (PID) meetings under the Veterinary Innovation Program (VIP) may be requested; for those wishing to pursue ‘dual species product approval', i.e., using animal data for regulatory approval for both the veterinary and human product, it may be helpful to meet with both Centers early in the process. For veterinary drugs, clinical studies are not broken into phase 1, 2, and 3 studies as in human medicine. **(B,C)** Including appropriate naturally occurring/spontaneous animal models in the human drug investigation process (T0.5) can better inform whether proceeding to human clinical studies is worthwhile and, with planning, can not only save time and money but may lead to dual species product approval without significant extra investment. Once clinical studies have been completed, human drugs require additional submissions such as a Biologics License Application (BLA) or New Drug Application (NDA) for FDA review; veterinary products require submission of a New Animal Drug Application (NADA). Once a product receives FDA approval, post-approval monitoring must be performed to ensure continued safety and efficacy. For more information see: Lee MH, Au P, Hyde J, et al. “Translation of Regenerative Medicine Products into the Clinic in the United States: FDA Perspective.” Translational Regenerative Medicine, edited by Anthony Atala and Julie Allickson, Elsevier Inc., 2015, 49–74.

First approved by the U.S. Food and Drug Administration (FDA) in 2017 to treat B-cell acute lymphoblastic leukemia (B-ALL) and diffuse large B cell lymphoma (DLBCL), chimeric antigen receptor T cells (CAR-T) therapies are one of the most promising new treatments for cancer (21, 22). CAR-T cell therapies use the patient's T cells modified by gene therapy to express a recombinant receptor that allows targeted cytolysis. Based on the positive and negative experiences of using CAR-T cells, other specialized immune cells, such as natural killer (NK) cells, are also being evaluated as novel cancer treatments. Despite their promise, therapeutic obstacles and optimal management of significant side effects for these therapies remain issues to overcome (21, 22). Although rodent and primate models may be critical in initial development, these preclinical models fail to capture some critical aspects of human disease and experiences; spontaneous canine cancers complement these models in testing and optimizing the safety and efficacy of therapies including feasibility, toxicity profiles, immune correlates, and outcomes (21, 23). In doing so, these therapies can provide new treatment options for canine patients and can lead to dual species product approvals, i.e., using the animal data to support “go/no-go” decisions and regulatory approval of both the veterinary and human product (Figure 2). Canine CAR-T cells have been developed by several laboratories and have been used in a small pilot study in dogs with advanced DLBCL (22–24).

### Pluripotent Stem Cells

Pluripotent stem cells (PSCs) can be maintained *in vitro* indefinitely under the appropriate culture conditions and give rise to all somatic and germ cells within the adult organism. The most stringent biologic assay of pluripotency is the tetraploid (4N) complementation assay in which putative PSCs are injected into a 4N blastocyst embryo that is then transplanted into a surrogate mouse mother (25). The pup that will be born will be composed of the stem cell progeny cells exclusively. Human PSCs cannot be tested as such due to ethical reasons and are typically tested for their ability to form teratomas once injected into an immunodeficient mouse recipient, proving the PSC capacity to form tissues of all three embryonic layers: endoderm, mesoderm, and ectoderm. PSCs were originally isolated from the inner cell mass of the blastocyst embryo, but in 2006 a method to reprogram adult, somatic cells into embryonic stem cell (ESC)-like cells was discovered (26). These so-called induced pluripotent stem cells (iPSCs) have revolutionized the field of regenerative medicine and personalized medicine because this technology has enabled the generation of autologous synthetic replacement tissues to cure numerous devastating diseases.

Research of veterinary species-specific PSC biology is in its early stages, and species-specific regulators of pluripotency are poorly understood (27). While several groups have reported on the generation of canine (28–31), feline (32) and equine (33, 34) ESCs and iPSCs, robust and reproducible cell lines and protocols are still elusive. A pioneering clinical trial investigating the use of canine iPSC-derived neuronal progenitor cells for chronic spinal cord injury in two pet dogs was recently published (35). Findings indicate that transplanted progenitor cells did not form a tumor; however, no clinical or electrophysiological improvements were noted on follow-up examinations.

### Organoids

Organoids are *ex-vivo* generated 3 dimensional (3D) cell structures that resemble an organ structure and cellular complexity (36). They have recently become of interest to the field of regenerative medicine as they could potentially mimic the organ of interest *in vitro* better than traditional 2D culture systems. These systems are preferred because 2D systems do not accurately reflect the response of organs to therapies or represent the safety or “tolerability” of certain pharmaceuticals (37). If stem cells can be utilized to form the organ of interest through stimulation in a microniche environment, which forms the tissue that composes the organ, more efficient and affordable studies can be done to determine the efficacy of treatments. Several groups have produced organoids from veterinary species, such as feline liver organoids to model hepatic steatosis (38), canine urinary bladder organoids to study muscle-invasive bladder cancer (39), and others (40).

### Exosomes/Extracellular Vesicles

Extracellular vesicles (EVs) are secreted by most cell types, and their function is in facilitating intercellular communication. They include exosomes, microvesicles, and apoptotic bodies, which have important therapeutic potential in controlling inflammation, enhancing regeneration, and repairing injured tissues (41). EVs derived from stem cells most likely have the same functions as the stem cells but avoid issues of potential immunogenicity that are often perceived as important in cellular therapies. EV's have been studied in many applications such as regenerative therapies, drug delivery, and immunomodulatory therapy (42). For example, EVs from canine AD-MSCs have been investigated in a mouse model of IBD, which spontaneously affects humans, dogs, cats, and horses (43).

## Cell Processing

Since every part of cell handling and processing can affect cellular phenotype, it is critical that standardized, detailed protocols are developed. Such protocols allow accurate assessment and design of comparative studies, which can help explain variable and unexpected outcomes and direct future studies, best practices, as well as scaled-up manufacturing. It is critical to acknowledge, identify, and understand cellular differences between species in order to develop species-specific cell processing protocols. Key variables include media (incl. fetal bovine serum (FBS)), oxygen, pH, cell culture substrate, cell seeding density, passaging frequency, harvesting, preservation, and distribution chain methodologies. This in turn highlights an important caveat with regards to the One Health approach in that one cell type working in one animal species may not be directly translated to a similar human type or a human condition. While all model systems have inherent limitations, the incorporation of naturally occurring disease in veterinary species along with traditional pre-clinical animal models of disease into a novel translational bio-medical research paradigm, may increase the predictive value of such data and its applicability to human medicine (44, 45).

### Culture Conditions

The impact of culture conditions on cell growth and function cannot be overstated. FBS is highly variable in content and potency from lot to lot, and its use can result in variability in culture outcomes, may be a cause of adverse reactions *in vivo* and transmission of infectious agents, and has raised animal welfare concerns (46, 47). For these reasons, serum-free and xeno-free media are used for cell processing of human cells used in clinical studies. The development of species-specific, serum-free media is needed in veterinary medicine. Oxygen tension is another aspect of cell culture, which has not received the same attention in veterinary cell processing protocols as it has in human protocols despite the role it plays in cell health and phenotype. Ambient air oxygen tension is hyper-physiologic and has been associated with reduced potency and increased cytogenetic abnormalities (48).

### Fresh vs. Cryopreserved

The utility of freshly harvested cells compared to thawed cells in regenerative applications remains a topic of debate. Several studies have shown that cryopreservation impacts cell function even when cell viability is high and surface markers are preserved (49, 50). The significance of the effects of cryopreservation may depend on the cryopreservation methods used, the cell application/desired outcome, and the species. The method details should be disclosed in publications, and viability, as well as functional assays, should be considered to assess the cells used in clinical trials.

### Manufacturing and Shipment

Practical manufacturing of cell therapy products will require scalable systems, which can be broadly classified as static or dynamic in nature. Static systems include T-flasks and stacked plate systems, which, while simple to operate, are labor-intensive and do not allow for great control of culture conditions. Alternatively, dynamic systems, including bag bioreactors, stirred suspension, and vertical-wheel bioreactors, are much more amenable to scale-up and automation. Shipment, which includes the shipping container and suspending agent as well as temperature, time, and movement, can similarly significantly affect cell viability, sterility, and function. Methods to test and ensure cell viability, quality, and sterility need to be implemented for all cell types and species as studies have previously noted significant variability in cell viability that likely affected study outcomes (51). An additional need and challenge is to investigate sustainable methods of providing cell therapies for all researchers and manufacturers in the field: decreasing the environmental impact of cell therapies increases the potential for their use, helps decrease the significant cost of providing these therapies, and decreases the counter-productive negative health impact these therapies have on the intended recipients.

## Quality Control

Similar across all species and applications, effective, large-scale use of cell therapies requires quality controls similar to other drug products but with some additional challenges. Quality control must be assessed from the early stages with screening of donors, through careful monitoring during processing, and ultimately with performance, sterility, potency, and functional assays of the final, delivered product. Donor screening is dependent on species and risk assessment and can draw from experience and techniques used for safe blood transfusion and organ transplantation to decrease spread of infectious disease and to identify optimal cell donors. Specific cell therapies may require additional donor screening techniques, such as testing for chromosomal abnormalities in PSC lines, to determine maximum efficacy (52). Sterility can be assessed by direct observation of cell morphology, growth characteristics, and visual presence of infectious organisms as well as culture at various stages in product development including the final product at the time of delivery. However, challenges exist regarding the need for reliable, rapid microbial testing methods to allow safe and timely product release (53). Challenges also exist in determining the best methods for assessing cell quality, function, and potency. Validated, feasible, and clinically-relevant assays are needed and may have to be product-specific considering the variability noted in tissue type, species, and disease application (54). Continued evaluation of the target and off-target effects and mechanisms of action of individual cell therapies are needed to provide additional information to evaluate short- and long-term safety and will greatly increase the potential for cell product approval.

## Cell Delivery

Cell delivery, survival, integration, and functionality are all critical in the long-term effectiveness of cellular therapies. The first decision is the administration route, which can be systemic or local. Systemic delivery has the advantage of being easier, but cells will be transported non-specifically to many areas of the body unless they are modified to home to specific locations. Alternatively, cells may be delivered directly to the desired site of action: injected into a defect (i.e., into a tendon or ligament defect where there is a core lesion and in which case there may be up to 95% cell retention) or transplanted as either a single cell suspension, as suspended cell aggregates, as micro-encapsulated clusters, or as tissue-engineered constructs. In the case of suspended single cells or aggregates, the viscosity, composition, and temperature-dependent behavior of the substrate can be manipulated to ensure fast delivery and high viability. In the case of encapsulated or tissue-engineered cell delivery, the physical, chemical, and structural properties of the biomaterial can be tailored to ensure the correct mechanical, chemical, and biological functioning of the cells/tissues. The size of companion animals allows investigation of all translationally applicable methods of cell delivery, which is not always feasible in preclinical laboratory animal models.

There has been growing interest in recipient characteristics and the ways that the variations in major histocompatibility complexes (MHC) may affect the survivability of cell products when delivered as therapies into several species (55, 56). Recent literature insinuates that repeated injections of MSCs of differing haplotypes may determine the efficacy of treatments (57). More research in this area is forthcoming.

## Immunogenicity Aspects of Cell Therapy

Immune recognition and subsequent destruction of allogeneic cells administered for therapeutic purposes is a topic of great interest in the regenerative medicine field. While allogeneic MSCs were considered to be immune-privileged, numerous pre-clinical, veterinary and human clinical studies have demonstrated that while MSCs employ multiple immune-evasive mechanisms, administered MSCs induce an immune response that is, at least partially, responsible for the lack of long-term engraftment (58, 59). Specifically, several groups demonstrated the formation of alloantibody in multiple species including cats, horses, pigs, macaques, rats, and humans in response to systemic infusion of allogeneic MSCs (58, 60, 61). However, the significance of such antibody development is currently unknown with regards to clinical outcome as patients demonstrate various clinical improvement despite antibody development and repeated dosing. The innate immune system, which has a key role in the initiation of the adaptive response, is further activated by the administration of the allogeneic MSCs (62, 63). The decreased immunogenicity of allogeneic MSC is driven by multiple mechanisms. MSCs express low levels of MHC class I and no MHC class II molecules when not activated. Moreover, human MSCs also express HLA-G, a non-classical MHC molecule that suppresses effector leukocyte function and was initially described in placental trophoblasts as a key player in maternal immune tolerance (64, 65). Moreover, MSCs secrete numerous paracrine factors (e.g., IDO, NO, PGE_2_, TGF-β, PD-L1 etc.) that shift classical monocytes to an immunomodulatory phenotype, suppress effector T cell activation and proliferation, and promote the differentiation of T regulatory cells.

While MSCs treatment delivers a therapeutic benefit in the absence of long-term engraftment (likely due to paracrine mechanisms), immune tolerance that enables long-term engraftment is critical for the transplantation of iPSC/EC-derived cells and tissues from mismatched donors. When human iPSCs were initially reported in 2007, hope for personal regenerative medicine application was on the horizon (26, 66). However, autologous iPSC treatments are not feasible at this time due to the high costs associated with the creation, characterization, and validation of iPSC lines from individual patients. As such, much effort is invested in efforts to create universal off-the-shelf iPSC-derived grafts. Such efforts include the creation of cryo-banks for HLA-homozygous iPSC lines to enable the clinical use of MHC-matched iPSC-derived grafts (67, 68). Efforts to create a universal iPSC line *via* genetic editing are further underway. Specifically, most research is targeting the deletion of MHC I to prevent cytotoxic CD8 T cell-mediated toxicity and at the same time to avoid NK attack that is driven by the lack of MHC I expression. Deletion of β2M and CIITA genes to prevent MHC-I expression and the induction of NK inhibitors such as HLA-E and CD47 have been reported (67, 69–71). Companion animals such as dogs, cats, and horses may represent a very attractive platform to study the immune compatibility of genetically modified universal iPSCs given the presence of well-defined breeds along with a more outbred population.

## Current Veterinary Clinical Trials Using Cell-Based Therapies

While numerous peer-reviewed manuscripts describing the use of MSCs in various veterinary clinical trials have been published, the Center for Veterinary Medicine (CVM) at the FDA has not yet approved any MSC or other animal cell-based therapy product for clinical use at the time of manuscript preparation (March 2021). Readers are referred to an informative recent paper reviewing veterinary clinical trials in the field of regenerative medicine (6). Currently, no central, searchable system exists for veterinary clinical trials. Animal studies are not listed on clinicaltrials.gov, and there is no requirement to publicly list animal clinical studies. It is therefore difficult to gain an overview of the field. Of note, the American Veterinary Medical Association (AVMA) operates an Animal Health Studies Database (AAHSD) that allows investigators and the general public to search for veterinary clinical trials, though trial registration is voluntary (https://ebusiness.avma.org/aahsd/study_search.aspx). A search *via* this database at the time of publication yielded no active studies and nine completed studies in the field of regenerative medicine. The FDA has recently launched a webpage where animal studies using cell-based products filed with FDA CVM can be listed (https://www.fda.gov/animal-veterinary/development-approval-process/clinical-field-studies-animal-cells-tissues-and-cell-and-tissue-based-products-actps). Participation is voluntary and is provided as a service by FDA to help facilitate study enrollment for clinical trials with an active FDA file that is in good standing. Other regulatory bodies are encouraged to provide similar listing services to aid in enrollment of clinical trials as well as providing an overview of the field. Active discussions focused on the development of veterinary cell-based therapy registries to track study information are ongoing.

## Effective Use of Animals Models

Animal models of disease have had an undeniable contribution to human research, providing significant contributions to medical understanding and advancement and preventing potential human harm. However, preclinical animal research has an unpredictable translation to humans, which raises ethical concerns as well as represents a large use of resources with no measurable benefit. Robinson et al. noted three areas of concern with animal models of disease: study design and data analysis, inherent heterogeneity of animal and human subjects, and the translation of preclinical animal trials to human clinical trials (72). Several other papers have highlighted similar concerns including issues with induced animal disease models and concerns over the impact of captivity on study results (73). As noted above, natural animal disease models in companion animals can overcome many of these concerns, including providing beneficial treatment to the animals themselves, but one would undermine their value by assuming they are without limitations. There are generally four options for disease models available today: human subjects research, induced disease models, artificial models, and naturally occurring animal disease models. Each of these models has its strengths and weaknesses, which can vary for the disease being studied. For example, spontaneous disease models can have increased variability, require longer study time, and, in the case of regenerative medicine, there is known variability in cell function between species (74). To truly optimize resources and outcomes, veterinarians, physicians, researchers, statisticians, and regulatory agencies need to work together to define needs, characterize models, share knowledge and information, design strong and relevant studies, and correctly assess the study results. Ultimately, optimal outcomes may require combining several models in a thoughtful and coordinated fashion to create impactful, sustainable translational applications.

## Conclusions and Consensus Statement

The development of therapeutic cell products has unique challenges that require a non-conventional, translational research approach and regulation. Specific challenges that are unique to cellular therapy include cellular engraftment, biocompatibility, and graft vs. host immune response. Moreover, given the inherent capacity of stem cells to self-renew and differentiate, stem cell-derived cellular products present unique safety challenges with delayed neoplastic transformation as a primary concern. Transplantation of human stem cell products into animal models does not model host response or graft behavior, regardless of the integrality of the recipient immune response. Given the critical significance of host-graft immune compatibility, a novel approach is warranted in which not only the disease of interest needs to be modeled but also the candidate therapy/cells.

We propose a novel paradigm for translational research of cellular therapeutic products that integrates a selective and highly informed use of spontaneous disease in animals. Veterinary clinician scientists are motivated and trained to facilitate such a paradigm shift toward a One Health approach. Academic veterinary hospitals, centers for veterinary clinical trials, and basic science laboratories are primed to provide the knowledge, infrastructure, and skill required to design and successfully execute meaningful translational research projects.

Importantly, current funding allocated by the NIH and other medical research funding agencies for such translational research projects is insufficient to capitalize on the potential benefit to human and veterinary patients. Addressing the immediate and critical need for funding and regulatory agencies to endorse companion animal, translational cellular therapies and to provide competitive monetary support for translational medicine research teams rooted within veterinary sciences would provide the means to actualize the potential of cellular therapies.

## Data Availability Statement

The original contributions presented in the study are included in the article/supplementary material, further inquiries can be directed to the corresponding authors.

## Author Contributions

BA: study conception and design, financial support, writing of manuscript, figure design, and final approval of manuscript. TW: study conception and design, financial support, collection of data, writing of manuscript, figure design, and final approval of manuscript. TK, SV, DB, AW, LG, MK, and AK: study conception and design, collection of data, writing of manuscript, and final approval of manuscript. All authors contributed to the article and approved the submitted version.

## Conflict of Interest

BA serves on the scientific advisory board of Gallant, TK serves as the founder, CEO and CSO of eQcell Inc., LG is a shareholder of Advanced Regenerative Therapies, and serve on the advisory board of eQcell Inc. The remaining authors declare that the research was conducted in the absence of any commercial or financial relationships that could be construed as a potential conflict of interest.

## Publisher's Note

All claims expressed in this article are solely those of the authors and do not necessarily represent those of their affiliated organizations, or those of the publisher, the editors and the reviewers. Any product that may be evaluated in this article, or claim that may be made by its manufacturer, is not guaranteed or endorsed by the publisher.
